# Perceptual experience in somatosensory temporal discrimination is indexed by a mid-latency fronto-central ERP difference

**DOI:** 10.1038/s41598-025-91580-1

**Published:** 2025-03-05

**Authors:** Jona Förster, Till Nierhaus, Pia Schröder, Felix Blankenburg

**Affiliations:** 1 Neurocomputation and Neuroimaging Unit, Freie Universität Berlin, Habelschwerdter Allee 45, 14195 Berlin, Germany; 2 Berlin School of Mind and Brain, Humboldt-Universität zu Berlin, 10117 Berlin, Germany

**Keywords:** Electrical stimulation, Somatosensory awareness, EEG, Post-perceptual processes, Event-related potentials, Distributed source reconstruction, Consciousness, Cortex

## Abstract

The neural correlates of conscious somatosensory perception are usually investigated using threshold detection tasks. However, it is largely unclear how other aspects of conscious somatosensory experience, such as localization, discrimination, and identification, are processed in the brain. Here, we go beyond mere stimulus detection and analyze the EEG data of 34 participants to investigate the event-related potential correlates of somatosensory experience in a temporal discrimination task. We show that the perceptual experience of feeling one vs. two pulses for identical pairs of electrical stimuli is reflected in positive fronto-central ERP activity after ~ 150 ms, even when controlling for task-relevance and post-perceptual processes such as decision-making and response preparation. This effect is a modulation of an ERP component that peaks considerably later at 170 ms and in a different sensor region than the detection-related so-called N140, which was not modulated by our task. Distributed source reconstruction of the sensor-level effect suggested the contralateral primary somatosensory cortex as its origin. We therefore propose that conscious detection and temporal discrimination are likely to both involve early sensory areas but recruit different neuronal processes. Our result adds to the growing body of research investigating the mechanisms underlying different aspects of conscious experience.

## Introduction

The study of consciousness and its neural correlates (NCCs) has made huge strides in recent years. Event-related potential (ERP) research converges on the view that negativities over modality-specific sensory cortices around 140–220 ms after stimulus presentation, with the precise latency depending on the sensory modality, correlate with the onset (as opposed to the sustainment^1^) of conscious perception of stimuli, while the fronto-central P300 component is more likely to reflect post-perceptual processes^2^. This view is supported by results in the visual^3–9^, auditory^10–12^, and somatosensory^13–15^ domains.

While there is a wealth of literature investigating the neural correlates of conscious processing, attempts to track and distinguish aspects of conscious experience beyond stimulus detection—such as localization, discrimination, or identification—are relatively rare. Therefore, it is currently not well explored how these processes interplay with conscious detection. Bachmann and Aru^16^ have recently developed a terminology for different types of experimental paradigms to study the NCCs of conscious processes of varying complexity, ranging from detection over discrimination up to paradigms where only the interpretation of conscious contents changes. Existing results suggest that the NCCs can considerably differ depending on which aspect of consciousness is being investigated: for vision, recent studies found that the visual awareness negativity (VAN) was elicited specifically by conscious detection, but not identification of stimuli^17^ (recently replicated^18^); that VAN amplitudes were modulated only in a color, but not in an object category discrimination task (and vice versa for the P300)^19^; and that a perceptual task recruited more occipital resources (the likely origin of the VAN^9^) than a conceptual task in a MEG study^20^. However, such studies are very rare in somatosensory NCC research, where detection paradigms remain the standard^13–15,21–23^.

One way to make a step beyond simple detection paradigms is by implementing somatosensory temporal discrimination, a paradigm that has been used in different contexts in many previous studies^24–26^, summarized in a recent review^27^. In this paradigm, double pulses separated by varying interstimulus intervals (ISIs) are presented to participants in order to assess their somatosensory temporal discrimination threshold (STDT). However, what is usually investigated are aspects such as the effects of different pathologies on STDTs^27^, or the areas implicated in temporal versus spatial discrimination^26^. There is one recent MEG study of somatosensory temporal discrimination that investigated the difference between feeling one vs. two electrical pulses and reported an event-related field (ERF) modulation in anterior and somatosensory sensors ca. 140–170 ms after stimulus onset^28^. However, the main focus of that study was on pre-stimulus effects in the frequency domain, and the ERF finding resulted from the comparison of ISIs at 0 versus 100 ms.

Here, we employed a somatosensory temporal discrimination paradigm in order to study how the perceptual experience of feeling one vs. two electrical pulses in response to stimulation with physically identical double pulses at STDT is reflected in ERPs, and where this difference in conscious content is most likely cortically represented. Recent studies have shown that ERP components can be mistaken to reflect conscious processing when what they more likely reflect are differences in task-relevance or response requirements^2,6,15^. In our study we controlled for these post-perceptual processes by means of a matching task^29^, designed to balance task requirements and the behavioral relevance of stimuli. In brief, the same percept could lead to different match/mismatch responses (and vice versa, different stimuli could lead to the same response), thus equating the influence of additional (post-) perceptual processes across perceptual experience. Compared to no-report paradigms, the matching task has the advantage of yielding definite perceptual labels on a trial-by-trial basis, rather than forcing the researcher to rely on block designs^4,7,12^ or proxies for conscious perception such as pupil size^30^, while nonetheless achieving a rigorous control, in terms of balance rather than abolishment, over post-perceptual processes.

Thus, the aim of our study was to investigate with EEG whether and, if so, how the NCCs of perceiving identical pulse pairs above detection threshold intensity as either one or two pulses differ from the well-established NCCs of conscious detection of single pulses around detection threshold, while controlling for post-perceptual processes^13–15,31^.

## Materials and methods

### Participants

Participants were recruited from the student body of Freie Universität Berlin. All participants gave their written informed consent and declared to have had no physical or psychological illness, and to have been right-handed according to the Edinburgh Handedness Inventory^32^. Data were only recorded when participants had stable psychometric functions and sufficient task performance in a behavioral training session on a separate day prior to the EEG recording session, leading to thirty-nine participants who completed the main experiment (one quit the experiment half-way through). Of these, five participants were excluded from the analysis due to excessive noise in multiple channels (caused by problems with the reference electrode and/or excessive movement artifacts), resulting in thirty-four participants (20 female, 14 male, age range 18–43 years) that were included in the analyses. Participants received compensation in the form of either money or course credits. The study was approved by the local ethics committee of the Freie Universität Berlin (003/2021) and conducted in accordance with the Declaration of Helsinki.

### Stimuli

The stimuli were pairs of direct current square wave pulses with 0.2 ms duration that were delivered by a DS5 constant current stimulator (Digitimer Limited, Welwyn Garden City, Hertfordshire, UK), through adhesive electrodes (GVB-geliMED, Bad Segeberg, Germany) attached to the left wrist, targeting the participants’ median nerve. To determine the detection threshold, we employed a brief staircase procedure: starting from an initial intensity value of 1 mA, this value was increased by 0.1 mA until the participant reported to feel the pulse. The stepsize was then halved, and the intensity decreased by the new stepsize until the participant reported not feeling the pulse anymore. The stepsize was then again halved, and the intensity increased by the new stepsize, and so on for three up- and three down-progressions in total. Intensity was then set to two times the individual detection threshold to ensure that no pulse could be missed due to low intensity. The resulting mean intensity was 4.85 ± 0.92 mA (all descriptive statistics are reported as mean ± SD, except when otherwise noted). In a second staircase procedure, an initial value for the individual STDT was determined. This procedure comprised the presentation of double pulses in three ascending and descending ISI series that were stopped when participants’ percept switched from one to two pulses or vice versa. The mean of these stopping points was 43.19 ± 31.53 ms. Starting from these values, participants’ psychometric functions for two-pulse temporal discrimination were estimated: participants received 15 ISIs (20 repetitions per ISI, leading to 300 trials in total over the course of ca. 9 min), linearly spaced around their initial discrimination threshold as the central of the 15 ISIs, and always beginning at 2 ms as the lowest ISI. The value of 2 ms was chosen to preclude the possibility of inducing the percept of a single high-intensity pulse, which could sometimes occur at ISIs < 1 ms. After each stimulus pair presentation, participants were required to respond via keyboard whether they had felt one or two pulses. A logistic function with two parameters (STDT and slope at STDT) was then fitted to the data (estimated 1%, 50%, and 99% discrimination thresholds: T01 = 2.45 ± 19.64 ms, T50 (STDT) = 40.51 ± 32.85 ms, T99 = 78.56 ± 62.04 ms; mean squared error across subjects: 0.0089 ± 0.0078). Based on these parameters, 10 different, individually calibrated and equally spaced ISI levels were determined and used in the main experiment. Based on previous studies^15,29^, the trial numbers within each ISI level followed a normal distribution, such that most trials occurred with ISIs close to the individual STDT (ISI levels 5 and 6: 32 trials/run each), and relatively few trials with ISIs far from STDT (ISI levels 1 and 10: 8 trials/run each). Note that in some participants, imperfect behavioral responses during the calibration phase led to a negative T01, as the proportion of “felt as one” trials at low ISIs and/or the proportion of “felt as two” trials at high ISIs was sufficiently below unity for the logistic fit to predict a negative T01. In these cases (9 in total), the minimum possible ISI for the main experiment was set to 1 ms, the maximum ISI was reduced by a proportional amount, and the remaining ISIs were shifted symmetrically toward the STDT, maintaining equal spacing. In theory, this procedure could have led to incomplete sampling of the psychometric function in the main experiment, in which case the participant would have been excluded. However, this never occurred in practice, and the psychometric functions of these nine participants in the main experiment were not compromised. Importantly, even though we were mainly interested in the near-STDT trials, we designed the experiment to include ISIs from the entire psychometric function for several reasons. First, sampling the entire range of ISIs served to maintain participants’ attention and interest in the task and prevent them from mere guessing. Second, it enabled us to identify slight shifts of the discrimination threshold over the course of the experiment and to monitor task performance. Third, it allowed compensating for such shifts. Stimuli were presented in MATLAB 2012a (The MathWorks) via the Psychophysics toolbox^33^.

### Experimental design

**Fig. 1:**
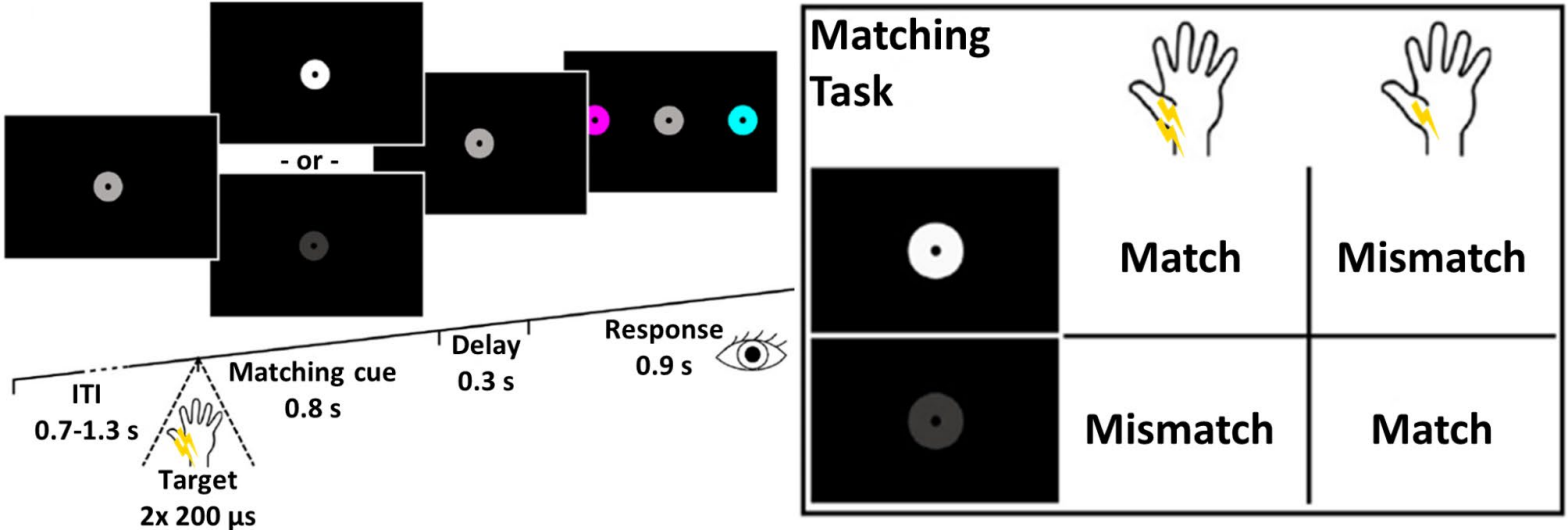
Experimental Design. After a jittered ITI, participants received a pair of electrical pulses to the left median nerve presented at twice the individual detection threshold intensity, with 1 of 10 ISIs separating the pair’s constituent pulses. Simultaneously, the gray fixation disk turned into a visual matching cue by changing its brightness, signaling either two pulses (white) or one pulse (dark gray). Participants then compared their percept (one vs. two pulses) to the visual cue and decided whether their somatosensory experience matched the meaning of the cue (according to the box on the right side of the Figure). Following a brief delay, participants reported their decision by saccading to one of two color-coded response cues presented at the sides of the screen.

After the calibration phase, participants performed two-alternative forced-choice somatosensory temporal discrimination via a visuo-tactile matching task^15,29^ (Fig. 1). Participants were seated in front of a computer screen and their eye movements were monitored by an eye-tracker (SMI RED-m remote, 120 Hz, Sensomotoric Instruments, Teltow, Germany). Every trial started with the appearance of a medium brightness gray fixation disk at the center of a black background. Participants were then presented with a pair of pulses separated by 1 of the 10 different interstimulus intervals (ISIs), and subsequently reported whether they had felt one or two pulses by comparing their experience with a visual matching cue that appeared simultaneously to the electrical stimulation. The matching cue consisted of a change in brightness of the gray fixation disk to either white or dark gray, signifying “two pulses” or “one pulse”, respectively. On each trial, this lead to one of four possible combinations of perceptual experience and visual cue (see box in Fig. 1, right): a “match” was constituted by (1) the participant feeling two pulses and observing the fixation disk change to white, or (2) feeling one pulse and observing the disk change to dark, while a “mismatch” was constituted by (3) feeling two pulses, but observing the disk change to dark, or (4) feeling one pulse, but observing the disk change to white. After each stimulus presentation, the fixation disk returned to medium brightness, and two colored response disks (magenta and cyan) appeared on the left and right sides on the screen (counterbalanced across trials). The colors coded for “match” and “mismatch” (magenta signified “match” in half of the participants, and “mismatch” in the other half), and participants responded by directing their gaze to the disk that corresponded to their experience (match or mismatch of somatosensory experience and visual cue). This match-mismatch procedure served to decorrelate temporal discrimination from overt reports, while using saccades instead of button presses to respond ensured that stimulus-evoked electrophysiological activity from somatosensory regions could not be influenced by response-related activity from the hand region of the adjacent motor cortex. When participants gave their response in time, the chosen response cue briefly increased in size; when they were too slow (> 0.9 s), the gray fixation disk in the center of the screen briefly turned red, signaling a missed trial. Individual trials were separated by intertrial intervals (ITIs) between 0.7 and 1.3 s.

### Experimental procedure and EEG recording

All participants performed a behavioral training session on a separate day prior to the experiment. When participants had stable psychometric functions and reached at least 90% accuracy in a task training run with only single pulses versus pulse pairs with very high ISIs (100 + ms), demonstrating comprehension of the task and ability to perform it correctly at low error rate, they were invited for the EEG recording. EEG data were recorded from 64 active electrodes positioned according to the extended 10–20 system (ActiveTwo, BioSemi, Amsterdam, Netherlands) with 2048 Hz sampling frequency. Vertical (vEOG) and horizontal (hEOG) eye movements were recorded with four additional electrodes. Twenty-seven participants completed seven blocks of 200 trials each (~ 10 min per block), resulting in a total number of 1400 trials per participant. The remaining seven participants completed only six runs (1200 trials), due to fatigue and/or eye tracker failures in one or more runs.

### Data analysis

#### Behavior

**Fig. 2:**
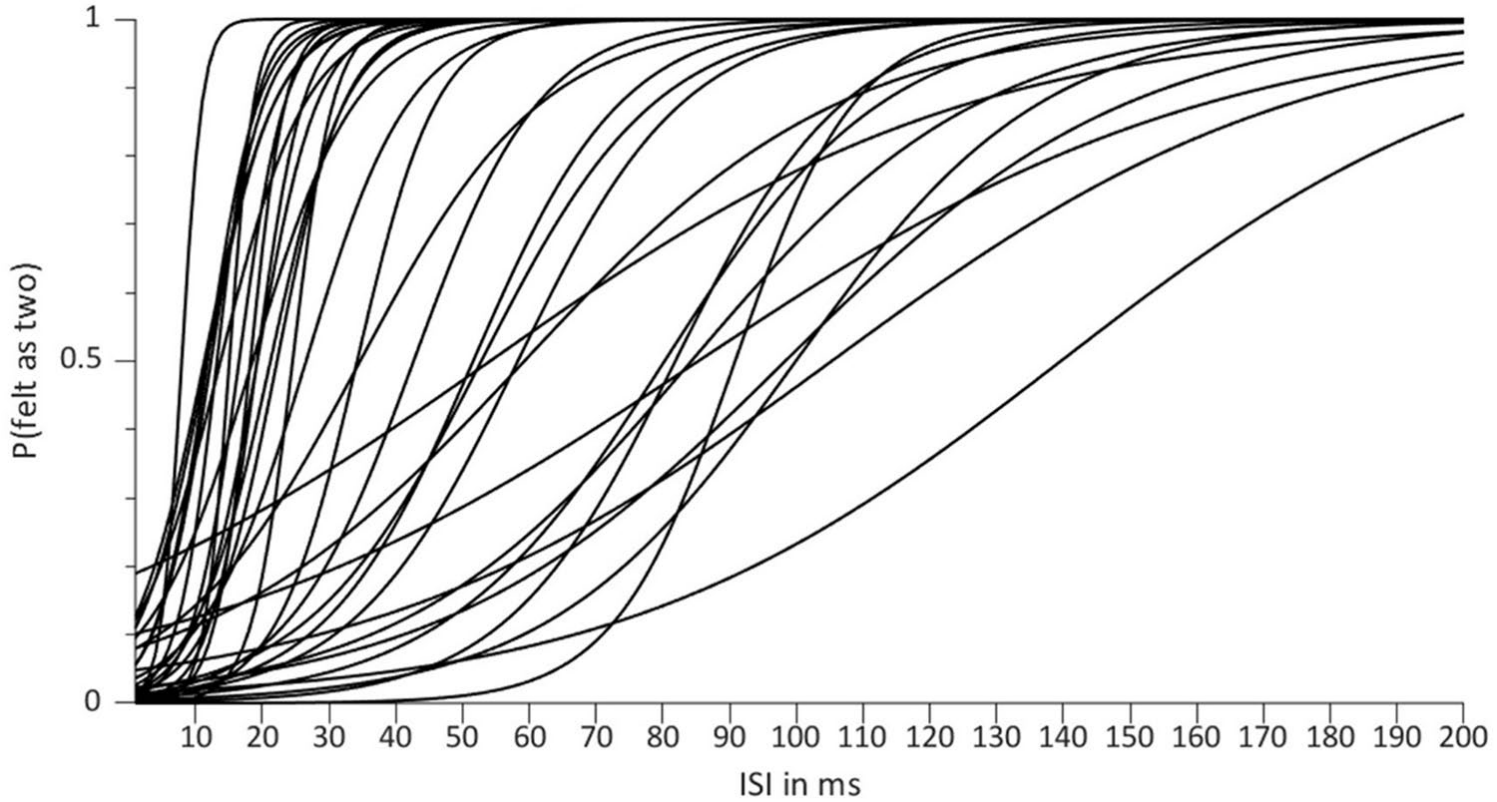
Run-averaged psychometric functions of all *n* = 34 participants included in the final analysis. The probability of feeling two pulses (as opposed to feeling one) is plotted as a function of ISI. The plot reveals considerable variability in discrimination thresholds (STDTs) and slopes across participants.

To visualize the distribution of STDTs across participants, we fitted logistic functions to the behavioral data of each run and averaged the estimated slope and threshold parameters for each participant, resulting in one mean psychometric function per participant (Fig. 2). Reaction time differences between “felt as one” and “felt as two” trials were tested using a Bayesian paired-sample t-test equivalent^34^; we report Bayes factors in favor of a difference (BF10). To test whether the matching task was successful in dissociating somatosensory temporal discrimination (felt one vs. felt two pulses) from overt reports (match vs. mismatch), we performed Bayesian tests of association (the Bayesian equivalent to a chi-square test^35^) for all participants and report Bayes factors in favor of the null hypothesis (BF01). Following the recommendations by Kass and Raftery, we consider 1 ≤ BF < 3 negligible, 3 ≤ BF < 20 positive, 20 ≤ BF < 150 strong, and 150 ≤ BF very strong evidence^36^.

#### EEG preprocessing

Preprocessing and all analyses were performed using SPM12 for EEG (www.fil.ion.ucl.ac.uk/spm) and custom MATLAB scripts. The data were high-pass filtered at 0.01 Hz, notch-filtered between 48 and 52 Hz, down-sampled to 512 Hz, and re-referenced to common average. Eye blinks were removed from the data using adaptive spatial filtering based on individual blink templates computed from the vEOG^37^. The data were then epoched from − 100 to 600 ms relative to the onset of the first pulse. Missed trials were excluded from analysis (2.1 ± 2.6%). All epochs were visually inspected for artifacts. Bad channels (containing > 20% bad trials) were interpolated either completely or on a run-by-run basis when only specific runs were affected. The remaining artifactual trials were removed (6.4 ± 5.1%). Finally, the data were low-pass filtered at 40 Hz and baseline-corrected using a baseline from − 100 to 0 ms. To keep the physical stimulus properties constant across perceptual experience categories, we based our analyses on the near-STDT trials. These were defined, for each given subject and run, as all trials from the ISI that was closest to the inflection point of the psychometric function, called “STDT trials” in the following (median number of ISI levels included per participant: 3, range ± 2; mean number of trials per participant: 185 ± 27). The preprocessed, epoched channel data were linearly interpolated into 32 × 32 × 359 voxel (scalp space  ×  intra-trial samples) 3-D images per trial. For visualization purposes, ERPs of “felt as one” and “felt as two” trials were computed for the STDT trials, and topographies were plotted using EEGLAB’s topoplot function^38^. These were then inspected for salient sensory-evoked potential components at characteristic time points.

#### Statistical analysis of the event-related potentials

All analyses were done within the SPM framework for M/EEG analysis. For the main analysis, first-level multiple regression models were then specified and estimated in SPM12, using “experience” dummy regressors for “felt as one” versus “felt as two” trials to regress, separately for each voxel, the EEG data over trials onto the outcome of perceptual experience. Without the inclusion of further regressors or covariates, this is mathematically equivalent to computing the ERPs of the two conditions. T-contrasts between the resulting beta parameters were then specified. At the second level, a t-test was run on the contrast images for “felt as one” versus “felt as two” trials, including a covariate representing each participant’s STDT. We report results with a cluster-defining threshold of ⍺ = 0.001 (uncorrected), using the random field theory-based approach to multiple comparisons correction (FWE) as implemented in SPM.

#### Source reconstruction

In order to estimate the most likely cortical sources of the observed sensor-level effect, we performed a distributed source reconstruction as implemented in SPM, spatially projecting the sensor data from scalp space into 3-D MNI voxel space. SPM12’s 8196 vertex template cortical mesh and BEM EEG head model were used to create a generic forward model and lead field. Group inversion using multiple sparse priors optimized with greedy search was employed to obtain group-wise source location and subject-specific dipole activation estimates^39–41^, separately for each condition (“felt as one”, “felt as two”). The results of the inversion were converted to images to bring them into the required format for SPM’s statistical machinery, and smoothed with a 4 by 4 by 4 mm full-width half-maximum (FWHM) Gaussian kernel. These images were then subjected to a group-level whole-brain analysis using an F-contrast between the two conditions’ difference to their respective baseline period activation. Because this was an unbiased analysis of a very subtle effect, we were primarily interested in the maximum intensity projection’s (MIP) location. We report uncorrected *p*-values for this analysis. Note that we disregarded clusters with an extent of k < 2 voxels (there was one single-voxel cluster).

## Results

### Behavior

Participants felt two pulses in 49.21 ± 10.04% (and one pulse in the remainder) of all trials (i.e., including all 10 ISIs), indicating that the experimental procedure worked as expected. As Fig. 2 shows, STDTs varied considerably (45.19 ± 35.32 ms, median = 30.38 ms, range = 131.66 ms). At STDT, we gathered 185 ± 27 trials per participant, which by definition divided into about 50% “felt as one” and “felt as two” trials, respectively. We found no evidence for a difference in reaction times between “felt as one” and “felt as two” trials (felt as one: 310.65 ± 48.19 ms, felt as two: 308.69 ± 48.4 ms, BF10 = 0.45). Bayesian tests of association provided positive evidence that the matching task successfully dissociated subjective perception from overt reports (4 < BF01 < 11 for all participants).

### Event-related potentials

**Fig. 3:**
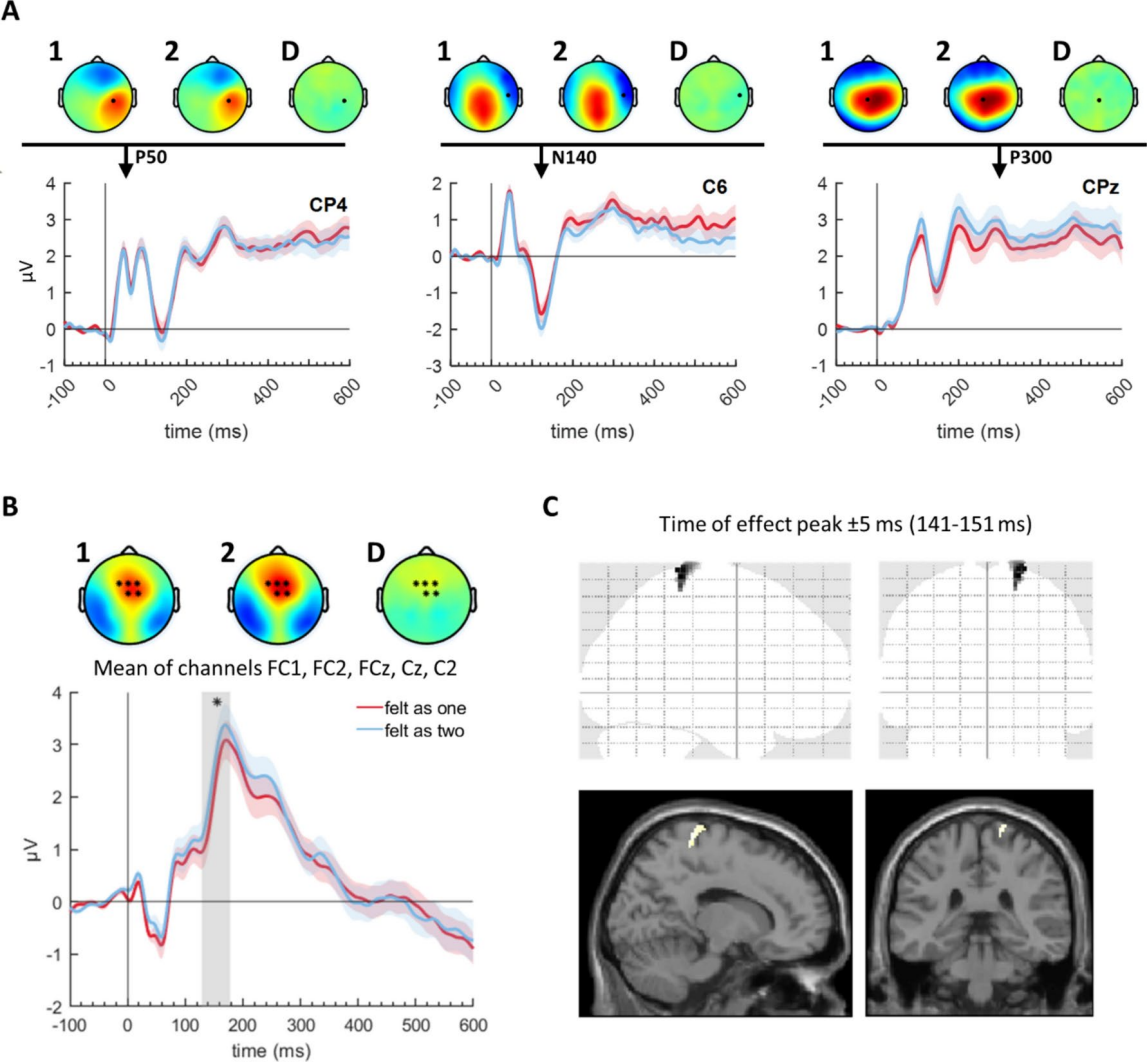
Grand-averaged ERPs of STDT trials, corresponding topographies, and source reconstruction, *n* = 34 (main analysis). (**A**) Grand-averaged ERPs at three electrodes selected for comparison with three components usually elicited by detection tasks (P50, N140, and P300), and corresponding topographies for “felt as one” (1), “felt as two” (2) and their difference (D). Colored shades denote standard error of the mean. Black dots represent electrode location. Note that the time point of interest for the N140 component was chosen as 120 ms, as that component peaked early in our data. (**B**) Grand-averaged mean ERP of the five electrodes that formed part of the cluster. Colored shades as in (*A*); gray vertical shade denotes time range of statistical significance; black asterisks denote electrodes that are part of the effect cluster. (**C**) 3D distributed source reconstruction at time point of sensor-level effect peak (146 ms) ± 5 ms. Upper panel shows sagittal (left) and coronal (right) MIP, lower panel shows the same results in SPM’s standard anatomical section template, centered on contralateral SI.

A first, purely descriptive inspection of the grand-averaged ERPs and topographies of the STDT trials of both conditions revealed potentials in electrode regions and at time points characteristic of well-known somatosensory-evoked potential components^42^, most notably positive deflections at 50 ms in electrode CP4 and at 300 ms in electrode CPz, and a negative deflection at 120 ms in electrode C6, shown in Fig. 3A. These components plausibly correspond to the P50^14,43^, N140^13,15^, and P300^15^.

### Statistical analysis

The main analysis of the entire sensor space revealed a significant fronto-central cluster ranging from 129 to 178 ms (*p*_FWE_ = 0.017, cluster size k = 618 voxels), and peaking at 146 ms (*p*_FWE_ = 0.033) in the vicinity of FC2. Further electrodes included in the cluster were FC1, FCz, Cz, and C2. This effect was a modulation of a positive component at electrode FC2 and neighboring electrodes peaking around 170 ms (P170, Fig. 3B). The inclusion of the covariate slightly enhanced the effect, but made no qualitative difference. To investigate a possible influence of shifts in the STDT during the experiment, we repeated the analysis, this time including a subject-level covariate encoding the ISI level of the STDT for each run. The results were qualitatively indistinguishable, yielding the same cluster around FC2, peaking at 146 ms (*p*_FWE_ = 0.04). Given the variability in STDTs across subjects, we performed another control analysis for two subgroups resulting from a median split based on the STDT (“low STDT” group: n = 17 participants, STDT < 30 ms; “high STDT” group: n = 17 participants, STDT > 30 ms). This analysis revealed that the effect of the main analysis was mostly driven by the low STDT group, in which the same cluster (albeit more lateralized, smaller, and later) was visible (*p*_FWE_ = 0.017, k = 489 voxels, 133–182 ms), again peaking at 146 ms, but just failed to reach significance at the peak level (*p*_FWE_ = 0.051). While the high STDT group on its own showed no significant effect, it thus still contributed to the overall effect both on the cluster level (greater cluster size in main analysis, k = 618 voxels) and on the peak level: no peak survived FWE-correction in the low ISI group, but two peaks did so in the main analysis comprising all participants.

### Source reconstruction

Based on the ERP results, we estimated the distributed source of the sensor-level effect in a time window of ± 5 ms around the peak. The source reconstruction resulted in a significant effect at MNI coordinate [18 -38 72] (*p*_*uncorr.*_ < 0.001), as part of a cluster located in contralateral primary somatosensory cortex (Fig. 3C). Note that this analysis was based on a small sensor-level difference and thus does not reach significance for a multiple comparisons correction of the whole brain. Although the results must therefore be viewed with the necessary caution, it is remarkable that it was possible to localize the cortical source to a single a priori plausible brain area in contralateral primary somatosensory cortex.

## Discussion

In this study, we investigated with EEG how perceptual experience in a somatosensory temporal discrimination task with electrical double pulses around STDT influences ERP components, using a matching task to control for post-stimulus differences in working memory and report requirements by dissociating these from perception itself.

Behaviorally, the variability in STDTs we observed in our study concurs with the considerable variability generally reported in the literature. Most studies stimulated the index finger, with thresholds ranging from about 20 to about 90 ms on average in healthy participants^27^. Studies that stimulated the left median nerve arrived at mean values comparable to our value of 45.19 ms (i.e., 50.27 ms^44^ and 45 ms^45^). Interestingly, unlike for “hits” and “misses” in an otherwise very similar detection task^15^, reaction times did not differ significantly between “felt as one” and “felt as two” trials in our temporal discrimination task (note that, to our knowledge, no other study so far has compared these reaction times). This indicates that task demands and subjective uncertainty are similar for the two perceptual outcomes in temporal discrimination, whereas they differed between “hits” and “misses” in detection tasks, even when controlling them rigorously^15^. This provides a first behavioral hint that conscious detection and temporal discrimination might constitute different processes.

We had hypothesized that if detection and temporal discrimination are two different processes, the difference in the conscious percept between “felt as one” and “felt as two” trials for identical physical stimulation would lead to an effect that is spatiotemporally different from the N140 component, which is usually elicited or at least strongly enhanced by the conscious detection of single stimuli^14,15^. Indeed, we found a modulation of a P170 component when the presented double pulses were perceived as two pulses rather than as a single pulse. The temporal specificity of this effect is corroborated by the fact that the high STDT subgroup, despite higher and more variable thresholds than the low STDT subgroup, contributed to the effect peak at 146 ms (rendering it significant in the main analysis comprising both subgroups), thereby indicating that the timing of the effect does not vary substantially as a function of STDT.

One plausible interpretation of this result is therefore that conscious detection constitutes a prerequisite for further processes operating on the “output” of detection. This supports similar results in the visual domain suggesting different neural correlates of conscious detection and identification^17,18^. There are several possibilities regarding the relationship between conscious detection and conscious temporal discrimination. Perceiving only one pulse may be a matter of fusing two percepts that have both been detected in the first place. Alternatively, it could simply be a matter of missing (i.e., not detecting) one of the pulses. The detection-related N140 component^14,15^ relative to the onset of the first pulse was present in both “felt as one” and “felt as two” trials, but was not modulated by this difference in perceptual experience. Hence, to the extent that our data permit such an assessment at all, it would presumably be the second and not the first pulse that is missed in this scenario. However, given that we stimulated at twice the individual detection threshold, it is unlikely that any pulses were missed merely due to low intensity in our study. An attentional effect similar to the one operating in the attentional blink phenomenon^46^, where the capturing of attention by the first pulse sometimes prevents the perception of the second, is conceivable. However, attention and working memory are thought to be largely irrelevant in somatosensory temporal discrimination^27^, and moreover, we controlled both processes with our matching task.

In the statistical analysis of the ERPs, we found a subtle but very robust fronto-central effect for the perceptual experience of one versus two pulses between 129 and 178 ms, peaking near electrode FC2 at 146 ms, as part of a P170 component. Our result is consistent with the location and time window of the effect reported in a recent MEG study, which had found an ERF amplitude increase in anterior and frontal sensors between 140 and 170 ms when participants perceived the double pulse as two pulses^28^. Note that this effect resulted from comparing the two experience categories for ISIs of 0 and 100 ms. The authors of that study only used this result to identify a time window of interest for a subsequent regression analysis of ERF power onto a particular ordering of pre-stimulus alpha power bins, and their result cannot be taken (and was not meant) as evidence that ERF amplitude in this window directly reflects perceptual experience, independent of physical stimulus properties. In contrast, our study shows that ERPs differ between “felt as one” and “felt as two” conditions even for identical stimuli (i.e., only pulse pairs at STDT).

A component similar to the P170 observed in our study has been reported for a single-pulse^47^ and a paired-pulse oddball paradigm^48^ (termed P150 in these studies), with amplitudes stronger for deviants than standards in both studies, and hence was interpreted as a somatosensory mismatch response. In the paired-pulse oddball paradigm, the deviant led to a stronger P150 even when it was felt as one pulse most of the time, and the standard was clearly felt as two pulses^48^. On the basis of the available results, it must remain open to what degree the P150/P170 is modulated by expectation (violation) in addition to perceptual discrimination as such. However, since our study was designed to yield an approximately equal amount of “felt as one” and “felt as two” trials distributed randomly over the course of the experiment, no strong expectations could have been built up. Therefore, mere expectation violation cannot explain our results.

In the terminology of Bachmann and Aru^16^, our use of the paradigm within the NCC context mostly falls in their category (2) (discrimination of perithreshold level stimulus objects, where “the same object appears subjectively with different qualities”, p. 2), but it also has aspects of their category (8) (ambiguous figures, where “different interpretations of conscious contents of the already consciously experienced stimulation”, p. 2) are possible. The most frequently studied paradigm in somatosensation is Bachmann and Aru’s category (1), i.e., the simple detection of single pulses. This paradigm commonly elicits P50^43^, N140^15^, and P300^3^ components. Interestingly, these components were elicited in our temporal discrimination task as well, but in contrast to detection studies, none of them were significantly modulated by the perceptual experience of feeling “one” versus “two” pulses. The detection-related N140 occurred already after 120 ms in electrode C6 in our study. Some early studies of attention in suprathreshold intensity regimes subdivided the N140 into an earlier and a later part^49^, or even viewed the N120 as a separate component, linking the N120 to somatosensory awareness and the N140 to spatial attention^50^. Like in our study, a recent continuous theta burst stimulation (cTBS) study of somatosensory temporal discrimination found an N120 component, and could show that cTBS over primary somatosensory cortex (SI) increased STDTs, whereas cTBS over secondary somatosensory cortex (SII) did not interfere with STDTs, but increased the N120 component of the sensory-evoked potential^44^. The short latency of this component in all these studies could plausibly be explained by the use of stimulus intensities clearly above detection threshold, which were therefore “easy” to detect. This is consistent with NCC research in vision; specifically, the VAN peaks earlier in masking studies (which use clearly detectable stimuli, followed by mask stimuli) than in studies using low-contrast stimuli, which are presumably harder to detect^9^. Together with the fact that the available evidence points to a strong involvement of SII in the generation of the N120/N140^13,22,29,44,50–53^, our results thus point to a dissociation or progression of the processes responsible for conscious detection and conscious temporal discrimination.

Our distributed source reconstruction of the effect peak time window (146 ± 5 ms) suggests contralateral SI (Fig. 3C) as the origin of the ERP effect. This is in line with both correlative^25^ and causal^44,54^ results showing that SI is the primary site implicated in somatosensory temporal discrimination (for a thorough recent review, see ref^27^). Importantly, these studies only showed a general involvement of SI in temporal discrimination, e.g., by showing that STDTs shift when SI is lesioned or manipulated, and also implicated other areas like the right pre-supplementary motor area and anterior cingulate regions^26,27^. Our study is the first to show that the difference in perceptual contents (“felt as one” versus “felt as two”) is itself represented in contralateral SI. Interestingly, somatosensory studies employing a range of tasks involving both temporal and spatial integration of stimuli have shown that SI is also critical in percepts resulting from spatial illusions^55,56^, frequency discrimination^57^ and localization/identification^58^, but not necessarily in detection. Mechanistically, processing in SI relies in part on a highly sensitive network of inhibitory interneurons that regulates thalamo-cortical inputs^59^ as well as various cortico-thalamo-cortical^60^ and cortico-cortical loops^61,62^, and the inhibitory activity of this network plausibly influences not only the sensory processing of near-threshold stimuli^63^, but also somatosensory temporal discrimination^27,44,45,54^. It has been suggested that coordinated activity of the inhibitory network in SI determines the STDT, and that cTBS of SI delays STDT by reducing this inhibition, thereby increasing temporal uncertainty^44^. The higher P170 amplitude for “felt as two” trials in our data may thus reflect the effect of increased activity of inhibitory interneurons, leading to a sharpened pyramidal cell response that enables more precise temporal separation of the two excitatory inputs induced by the double pulse, and thereby to a conscious percept of “two pulses”. In contrast, when fewer inhibitory interneurons are active, or their activity is less coordinated, their sharpening effect on the pyramidal cells will be smaller, resulting in weaker dipoles that are more spread out in time, and consequently in a P170 that is less sharp and of smaller amplitude, indicating decreased temporal certainty that leads to a conscious percept of only “one pulse”. This mechanism could also underlie the recovery functions of mid-latency components^64^, while incomplete recovery of the refractory period of the initial SI response after the incoming peripheral thalamic activity volley (as reflected in the N20 component^65^) is unlikely to be responsible for the effect in our study, both because this effect occurred much later (even in relation to the second pulse in the majority of participants), and because perceptual discrimination of paired pulses has been shown to be possible already before the N20 has fully recovered^66^. One recent study found that the phase of pre-stimulus beta oscillations in SI influences perceptual outcomes^67^, in accordance with the “perceptual cycles” framework^68^: only when two pulses fall within two distinct perceptual (beta) cycles will they be perceived as two. When they fall within the same cycle of an ongoing beta oscillation, they will be perceived as one. While this explanation is in principle compatible with both the results of our study and the inhibitory account outlined above, the precise connections between them remain to be explored.

The P300 did not differ between conditions in our experiment. Given the subtlety of the P170 effect, it seems unlikely that a mere lack of power is responsible for this null finding in a large component like the P300 that usually shows massive modulations when the generating processes are subject to manipulation. As we had rigorously balanced working memory and report requirements between conditions, any P300 difference could with reasonable confidence have been attributed to processes involved in temporal discrimination. Our negative result instead indicates that the P300 is not a marker of perceptual discrimination, thereby adding to the recent flurry of results showing that the P300 is not a neural correlate of conscious perception, but reflects differences in task-relevance and post-perceptual processes^3–9,12,15^.

In conclusion, we have shown that the conscious perceptual outcome of somatosensory temporal discrimination is indexed by the modulation of a fronto-central P170 component, which is spatiotemporally separable from both the detection-related N140 and the post-perceptual P300, and thus presumably generated by a distinct process that is likely located in contralateral SI.

## Data Availability

Preprocessed data and the code necessary for reproducing the results will be shared upon reasonable request to the corresponding author.
